# *WBSCR* Locus: At the Crossroads of Human Behavioral Disorders and Domestication of Animals

**DOI:** 10.3390/ijms26178549

**Published:** 2025-09-03

**Authors:** Mikhail V. Shepelev, Olga I. Skobel, Tatiana T. Glazko, Dmitry V. Popov, Denis E. Vysotskii, Pavel G. Georgiev, Oksana G. Maksimenko, Gleb Y. Kosovsky, Yuliya Y. Silaeva

**Affiliations:** 1 Center for Genome Research, Institute of Gene Biology, Russian Academy of Sciences, 34/5 Vavilova Str., 119334 Moscow, Russia; maksog@mail.ru; 2 Afanas’ev Institute of Fur-Bearing Animal Breeding and Rabbit Breeding, 6 Trudovaya Str., Rodniki, 140143 Moscow Region, Russia; skobelolga@gmail.com (O.I.S.); tglazko@rambler.ru (T.T.G.); popov.bio@gmail.com (D.V.P.); visotskiydenis@mail.ru (D.E.V.); gkosovsky@mail.ru (G.Y.K.); 3 Department of the Control of Genetic Processes, Institute of Gene Biology, Russian Academy of Sciences, 34/5 Vavilova Str., 119334 Moscow, Russia; georgiev_p@mail.ru; 4 Core Facility Center, Institute of Gene Biology, Russian Academy of Sciences, 34/5 Vavilova Str., 119334 Moscow, Russia

**Keywords:** domestication of animals, Williams–Beuren syndrome control region, neurobehavioral disorder, *GALNT17*, *GTF2I*, *AUTS2*, autism spectrum disorder

## Abstract

Social interaction between the domesticated animal and the domesticator is one of the key features of the “domestication syndrome”. Recent research has identified genes in the *WBSCR* (Williams–Beuren syndrome control region) locus as significant contributors to social behavior in dogs. Large chromosomal deletions and duplications in the human *WBSCR* locus lead to the development of WBS (Williams–Beuren syndrome) and *WBSCR* duplication syndrome, respectively. Hypersociability is one of the key symptoms of WBS, while the duplication syndrome is manifested as an autism spectrum disorder (ASD). The data from both humans and dogs highlight the *WBSCR* locus as one of the key genetic determinants of social behavior in mammals. Several genes in the *WBSCR* are candidates for the regulation of social behavior in mammals including *GTF2I*, *GTF2IRD*, *AUTS2* and *GALNT17*. Here, we discuss the role of *WBSCR* locus in the regulation of social behavior in mammals including the recent data that highlight the importance of 3D genome alterations in this genomic region for both domestication of animals and development of neurobehavioral disorders in humans. In addition, we bring attention to the role of the poorly characterized *GALNT17* gene as a putative player in the development of ASD symptoms and in the regulation of social behavior in animals. We provide a brief summary of its known functions and propose the future research directions aimed at the elucidation of *Galnt17* involvement in the regulation of central nervous system (CNS) functions.

## 1. Genetic Basis of Domestication

Charles Darwin noted that domesticated animals have a distinctive and unusual set of hereditary traits that are absent in their wild ancestors [1]. The combination of these traits is called the “domestication syndrome” (a term previously used for domesticated crop plants) [2]. While many hypotheses focus on the mechanisms underlying specific economic traits (e.g., milk production in cows, wool quality in sheep, etc.), a recent explanation addresses traits common to all domesticated animals that allow them to live in captivity and interact with humans [3]. Namely, it was hypothesized that changes in activity of certain upstream genes in a genetic regulatory network lead to changes in the regulation of downstream genetic modules [4].

Research has shown that during the initial stages of domestication, reduction in animals’ stress during interactions with humans typically occurs through selection of animals with adrenal hypofunction and reduced stress hormone production [5]. However, the diverse phenotypic traits comprising the domestication syndrome cannot be explained solely by decreased adrenal function. Development of all traits of domestication syndrome is closely associated with neural crest cells (NCCs). These cells first appear during early embryogenesis at the dorsal edge (“crest”) of the neural tube and then migrate, giving rise to precursors of many cell types and tissues, such as cranial bones, adrenal medulla, pigment melanoblasts, odontoblasts [6,7,8]. Although NCCs are not direct precursors to any part of the central nervous system (CNS) or the adrenal cortex, they play an important role in the development of these tissues through post-migratory embryological interactions [9]. The hypothesis on the role of NCCs in domestication has received experimental support: accumulating evidence suggests that domesticated species have higher polymorphism of genes involved in the control of neural crest and its derivatives than closely related wild species. In particular, a study of 15 pairs of such species found that the number of codons with a relatively high ratio of nonsynonymous to synonymous substitutions was higher in domesticated animals compared to closely related wild species [10].

One of the recent studies proposes that the phenomenon of domestication is based on human intervention in the reproductive functions of both males and females during the process of domestication: limited interaction between males of the same species, limited pool of males participating in reproduction, selection of the most productive females in conditions of unlimited access to resources for feeding offspring and selection of females experiencing minimal stress when interacting with humans [11]. These interventions result in changes in the neural crest, since neural crest cells are involved in many processes during ontogenesis. This is consistent with earlier studies, which, in particular, showed that when backcrossing a domestic rabbit with a wild one, a seasonality of reproduction appeared in the second generation. This effect was not observed in the domestic rabbit [12].

Thus, despite the fact that the phenomenon of domestication has been known for thousands of years, its mechanisms have been studied rather poorly and there is no consensus on the genetic basis of this phenomenon. Meanwhile, the study of the genetic basis of domestication has recently acquired particular importance in connection with the pace and biospheric consequences of the development of agrarian civilization, the rapid reduction in biodiversity and growth of biomass of domesticated animals [13]. Therefore, the problem of clarifying the mechanisms of domestication is becoming more and more practically important, since the need for its management is consistently intensifying.

It is obvious that the genetic basis of domestication is polygenic and will differ depending on the tasks faced by the domesticator. The process of domestication involves taxonomic, ecological and cultural components that differ for each specific case. In general, in the case of successful domestication, the mutual benefit and adaptability of both the domesticated species and the domesticator increases [14]. In particular, it is possible to trace the parallel evolution of a number of genes in humans and dogs, which is most obvious in the genes responsible for food digestion, metabolism, neurological processes and the control of cell division, apoptosis and oncogenesis [15]. Based on the above, it becomes obvious that the process of domestication is regulated by incredibly complex genetic mechanisms. As a result, a variety of phenotypic characteristics of animals are changed and they are not limited to those selected by the domesticator. In the first direct multi-year experiment on the domestication of foxes, Dmitry K. Belyaev chose a decrease in aggression towards humans in a series of generations as a universal sign of domestication. However, after a number of generations, in addition to a decrease in the aggression, some other signs of domestication began to be detected in the offspring, such as a tail shape typical for dogs, drooping ears and a dog-like bark [16]. This means that, using classical selection methods, a domesticator can change only the complex of traits included in the domestication syndrome, but not its individual elements. Considering the modern possibilities of genome editing, the task of altering individual elements of the gene network regulating the entire complex of domestication syndrome is very attractive. It allows, on the one hand, to achieve a reduction in aggressiveness of domesticated mammals, and, on the other hand, to retain valuable economically significant phenotypic traits of wild animals (color and thickness of fur, tail shape, meat quality, etc.).

In this review, we will focus on the social behavior of animals as one of the crucial traits of the domestication syndrome. Recent genetic analyses of dogs and wolves—two related species that represent a domesticated species and its wild counterpart—have revealed an important role of the Williams–Beuren syndrome critical region (*WBSCR*) genomic locus in regulating social behavior in dogs [17]. Remarkably, the same genomic region is disrupted in Williams–Beuren syndrome (WBS), a human neurodevelopmental disorder characterized by hypersociability [18]. In addition, we will thoroughly discuss the emerging role of a *GALNT17* gene from the *WBSCR* locus in the regulation of social behavior in mammals. On top of that, we will highlight the effects of 3D genome alterations in the *WBSCR* locus on both domestication of animals and development of neurobehavioral disorders in humans. The genetic basis of animal domestication, particularly selection for enhanced sociability toward humans, provides a compelling evolutionary framework for understanding certain human neurodevelopmental diseases. We propose that in order to reduce the aggressiveness of domesticated animals towards humans, genes that are involved both in the process of domestication in mammals and various neurological or neurodevelopmental disorders in humans can be considered as possible targets for genome editing [19].

## 2. Genetic Basis of Behavioral Disorders in Human

### 2.1. The Genetics of Behavioral Disorders in Human

Social behavior in mammals is a complex phenotype regulated by multiple genetic pathways that have been conserved throughout evolution [20]. Social behavior of every individual in the population fits into a certain area of a sociability spectrum that could be presented as a normal distribution of behavioral phenotypes [21]. Behavioral disorders manifest with either hyposociability or hypersociability which are found at the extremities of the sociability spectrum [21]. Recent advances in molecular genetics, genome-wide association studies (GWAS) and functional genomics have identified numerous genetic loci that play critical roles in regulating various aspects of social behavior, from basic social recognition to complex behaviors [21,22]. Of particular importance for the regulation of social behavior are genes from oxytocin–vasopressin neuropeptide system: oxytocin receptor gene (*OXTR*, located on human chromosome 3p25.3) and arginine vasopressin receptor genes (*AVPR1A* on 12q14.2 and *AVPR1B* on 1q32.1) [23]. Other examples of functional gene groups include, but are not limited to, the following: neurotransmitter receptors (dopamine D2 receptor gene (*DRD2*, 11q23.2)) [24]; synaptic adhesion and scaffolding proteins (postsynaptic density protein PSD95 (*DLG4* gene on 17p13.1)) [25,26]; neuroligin gene family (e.g., *NLGN3* on Xq13.1) [27]; SHANK gene family (*SHANK1* on 19q13.33, *SHANK2* on 11q13.3, *SHANK3* on 22q13.33) [28]; and many others. Besides individual genes, there are multiple genomic loci affected by chromosomal aberrations (deletions, inversion, duplication) that lead to behavioral disorders in human. These include 1q21.1, 7q11.23, 15q11-q13, 16p11.2 and many others [21,22].

Autism spectrum disorder (ASD) refers to a group of heterogenous neurobehavioral disorders which are characterized by impaired social communication (hyposociability) and stereotypic behavior. Genetic defects in ASD include chromosomal aberrations (deletions, duplication) or point mutations in individual genes. Currently, over 800 individual genes and dozens of genomic loci with chromosomal rearrangements are associated with the development of ASD in human [22,29,30]. One of these loci is the *WBSCR* on chromosome 7 in human that is implicated both in the development of several ASD syndromes as well as the neurodevelopmental hypersociability disorder called WBS.

### 2.2. The Williams–Beuren Syndrome Critical Region (WBSCR) and Behavioral Disorders

**Figure 1:**
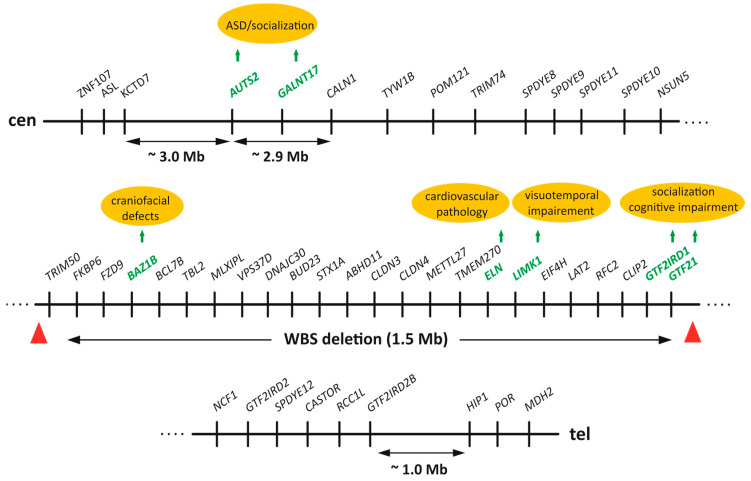
Scheme of the human Williams–Beuren Syndrome Critical Region (*WBSCR)* genomic locus. All protein-coding genes from *AUTS2* to *GTF2IRD2B* in the ~5.26 Mb region are shown in a centromere (cen)-to-telomere (tel) orientation. Notably, more than half of the ~5.26 Mb genomic range (~2.9 Mb) is occupied only by three genes: *AUTS2*, *GALNT17* and *CALN1*. The commonly deleted region in Williams–Beuren Syndrome (WBS) patients (1.5 Mb) is marked by red triangles and include genes from *TRIM50* to *GTF21*. Several flanking genes on the centromeric (*ZNF107*, *ASL*, *KCTD7*) and telomeric (*HIP1*, *POR*, *MDH2*) flanks of the *WBSCR* locus are also shown. Arrows indicate the approximate distance between genes. Several genes with known genotype–phenotype associations are highlighted in green. Scheme is not drawn to scale.

WBS (OMIM: #194250), that is sometimes called “hypersociability syndrome”, is caused by a 1.55–1.83 Mb deletion in the *WBSCR* located in 7q11.23 [18,31]. In 95% of patients with WBS, a 1.5 Mb hemizygous deletion encompasses 24 genes from *TRIM50* up to *GTF2I* (Figure 1); 4% of patients carry a 1.8 Mb deletion that includes two additional genes (*NCF1* and *GTF2IRD2*) and 1% of patients carry other deletions of variable size [32]. In populations, WBS occurs with a frequency from 1:7000 to 1:20,000 of newborns, according to different estimations [33]. It is believed that a high rate of chromosomal rearrangements in this locus is due to the presence of region-specific repeats (low-copy repeat elements, LCRs), that flank the deleted region [34] and mediate non-allelic homologous recombination during meiosis [35,36,37].

Patients with WBS are characterized by a developmental delay, intellectual disability, hypersociability, hyperacusis, anxiety, defects in visuospatial constructive cognition, speech delay, craniofacial anomalies and some other somatic symptoms [18] and these defects are accompanied by the morphological changes in the brain structure [38].

Genetic alterations in the *WBSCR* are not limited to deletions but also include duplications and triplications. The Williams–Beuren region duplication syndrome (WBDS; OMIM: #609757) is caused by a hemizygous duplication of the region that is usually deleted in patients with WBS and leads to the development of ASD [39,40] that is manifested with intellectual disabilities and speech delay [21,41]. Triplication syndrome is a rare event and is characterized by a phenotype that is similar to duplication syndrome but with more severe symptoms [42]. Notably, mice bearing the WBS deletion replicate most of the human symptoms, including hypersociability, thus indicating the evolutionary conserved features of the *WBSCR* locus [43].

Hypersociability phenotype of patients with WBS indicate that some genes in this locus are associated with social behavior and hence might also be involved in the evolution of social behavior in animals. Analysis of individual genes in the deleted region revealed that *GTF2I* and its paralogue *GTF2IRD1* might be the primary candidates responsible for the hypersocial phenotype of WBS patients. Loss of these genes is associated with intellectual disability, anxiety and impaired social behavior [18,41,44,45]. It has been shown that changes in the *GTF2I* expression level can affect the balance of excitation/inhibition of cortical neurons [46], that is, an agreement with multiple evidences pointing out such balance as the basis of the socialization network [47,48].

*GTF2I* gene encodes transcriptional factor II-I (TFII-I). In mice the peak expression of *Gtf2i* is detected during prenatal and early postnatal development [49]. Gtf2I regulates cell cycle [50], embryonic development [51], coordinates activity of multiple transcription factors [52]. Reduced levels of Gtf2I lead to mitochondrial dysfunction as manifested by impaired fission/fusion, autophagy and mitophagy [53].

Experiments using cortical organoids obtained from induced pluripotent stem cell (iPSC) lines have shown that chromosomal rearrangements in the *WBSCR* locus lead to disturbances in the proliferation and maturation of neurons, and such disturbances are reproduced if the expression level of only one gene, *GTF2I*, is changed [41]. The idea of the leading role of the *GTF2I* gene in the formation of the pathological phenotype was confirmed using mouse model: an increase in the expression level of *Gtf2i* led to a disruption of social interactions [54].

In addition to neurobehavioral disturbances, deletions and duplications in the *WBSCR* lead to multiple somatic pathologies. The best studied example of genotype–phenotype relationship in the *WBSCR* locus is the deletion of *ELN* gene. *ELN* encodes elastin, a protein that forms polymers in the extracellular matrix. Deletion of *ELN* in WBS patients leads mostly to a stenosis of large arteria and hypertension due to the loss of vessel elasticity [55]. These data are supported by the mouse model of heterozygous *Eln* knockout [56].

The contribution of the majority of other deleted/duplicated genes to the development of WBS phenotype is largely unknown. There are limited data on the genotype–phenotype relationship for several commonly deleted genes. The *WBSCR* locus contains the *BAZ1B* gene (also known as Williams syndrome transcription factor, WSTF), which is involved in chromatin remodeling and is necessary for the correct migration of neural crest cells in vitro and in vivo [57,58]. It is believed that loss of *BAZ1B* is associated with the presence of craniofacial defects in patients with WBS [18].

Another gene in the deleted region, *LIMK1*, regulates the assembly and disassembly of the actin cytoskeleton and is associated with impaired visual–spatial cognitive abilities in people with WBS [59]. In *Limk1* knockout mice, a decrease in long-term memory is observed [60]. Input of several genes (e.g., *STX1A*, *MLXIPL, DNAJC30*) to WBS phenotype and other aspects of WBS are discussed in the excellent review by Kozel et al. [18].

Two other genes from the *WBSCR* locus, *AUTS2* and *GALNT17*, are not affected by common deletions/duplications in WBS/WBDS. These genes together with *CALN1* gene flank the commonly deleted region in WBS on the centromeric side (Figure 1). Notably, these three genes span 2.9 Mb in the genome that is longer than the 1.5 Mb deletion in WBS patients (Figure 1). Pathogenic structural variants in *AUTS2* gene include translocations, duplications, deletions or single-nucleotide polymorphisms (SNPs) and lead to the development of *AUTS2* syndrome [61]. Core symptoms in patients with *AUTS2* syndrome include developmental delay and intellectual disability as well as microcephalia, autistic symptoms, attention deficit hyperactivity disorder, craniofacial abnormalities and certain somatic pathologies [61,62]. These symptoms at least in part are replicated in *Auts2* heterozygous knockout mice [63].

*GALNT17* (*WBSCR17*) gene encodes *N*-acetylgalactosaminyltransferase enzyme of which cellular functions remain largely elusive. Several SNPs in *GALN17* gene are linked to the development of Parkinson’s disease (PD) [64], but no structural variants in *GALNT17* have been definitely associated with the development of neurobehavioral disorders in human. Loss of *Galnt17* in mouse leads to ASD symptoms, developmental delay and anomalies of cerebellum [65] (see below), thus placing *GALNT17* as an attractive candidate for further dissecting its role in the development of neurobehavioral disorders and domestication of animals.

The putative role of *Galnt17* as a driver of ASD symptoms was uncovered using a mouse model with reciprocal translocation between the fifth and eighth chromosomes [62]. The breakage point on the fifth chromosome is located between *Auts2* and *Galnt17* genes and translocation results in decreased expression of both genes. The phenotype of the animals is characterized by growth abnormalities (lower body weight and length), facial skeleton anomalies, behavioral, memory and learning disturbances, increased anxiety, decreased exploratory activity and decreased righting reflex [62]. Animals with a homozygous translocation were found to have an altered structure of the hippocampus and cerebellum. Some of the phenotypic manifestations are similar to *Sox1* gene knockout (KO) that is located on the eighth chromosome and is disrupted by the translocation, but most of the phenotypic features are likely associated with a decrease in the levels of *Auts2* and *Galnt17* transcripts. Many phenotypic features are characteristic of *Auts2* gene KO mice [66] and patients with *AUTS2* syndrome [61,67]. Notably, co-expression of *Auts2* и *Galnt17* was detected in most cell types in the brain thus allowing us to predict that these two genes are located within one TAD (topology-associated domain) and therefore their expression is also co-regulated.

Taken together, this growing body of evidence suggests neurobehavioral and somatic phenotypes that linked to genes in the *WBSCR* locus are attributed not only to changes in copy number of deleted/amplified genes but also to changes in the expression levels of flanking genes due altered 3D genome organization as a result of large chromosomal rearrangements in this locus. Recent findings both in mouse and dog models (see below) indicate that the input of the altered 3D genome in the *WBSCR* locus is indispensable for the social behavior of animals as well.

## 3. *WBSCR* Locus and Dog Domestication

Evidence for the possible involvement of the *WBSCR* locus in the domestication process was obtained by GWAS analyzing more than 48,000 SNPs in the genomes of dogs of various breeds and in the gray wolf genome. As a result, one of the SNPs associated with positive selection in domesticated dogs was identified near the *WBSCR17* gene [68]. Subsequent targeted analysis of more than 25,000 SNPs in a 5 Mb region of the *WBSCR* locus on chromosome 6 in dogs revealed 89 structural variants (SVs) in *GTF2I*, *GTF2IRD1*, *AUTS2*, *WBSCR17* (*GALNT17*), *GALNT9*, *CBX3*, *BAZ1B*, *NSUN5*, *POM121* and *STYXL1* genes. These SVs are retrotransposons and four of them are reliably associated in dogs with attention bias to social stimuli: two in the *WBSCR17* gene, and one in *GTF2I* and *GTF2IRD1* genes. Thus, for the first time, a relationship was established between structural variants in the *WBSCR17*, *GTF2I*, *GTF2IRD1* genes and hypersociability in dogs in relation to humans, which is one of the critical features of domestication syndrome [69].

Subsequent analysis of the molecular mechanisms that may underlie the changes in social behavior in dogs showed that retrotransposons near and in the *WBSCR17* (Cfa6.6 and Cfa6.7), *GTF2I* (Cfa6.66) and *POM121* (Cfa6.83) genes are hypermethylated, which leads to disruption of the expression of the *WBSCR17*, *GTF21I*, *LIMK1*, *WBSCR27*, *BAZ1B* and *BCL7B* genes [17]. Thus, positive selection for hypersociability towards the host in dogs is based on the presence of mobile genetic elements which are methylated to affect the expression profile of several genes in the *WBSCR* locus. In particular, the *GTF21I* and *WBSCR17* genes, which are associated with the regulation of social behavior. Importantly, it was uncovered that the presence of one transposon element (TE) affects the expression of several genes in the locus, indirectly indicating the involvement of distant cis-regulatory interactions between genetic elements in the *WBSCR* locus. This was confirmed in further work using the example of a TE insertion in 17th intron of the *GTF2I* gene as discussed below.

It was found that in dogs, the insertion of TEs into the *GALNT17* gene leads to increased learning ability and social activity, which is accompanied by a decrease in the transcription of this gene due to an increased methylation of the transposon insertion region [17,70,71]. Moreover, it was shown that the higher the number of TE copies, the higher the degree of hypersociability in the dog. Genotyping of dogs for the presence/absence of a retrotransposon insertion (Cfa6.6) in the *GALNT17* gene is proposed for use in selecting puppies for raising service dogs (guide dogs) [70]. Although these genes in dogs are not damaged by the deletion/duplication observed in humans with WBS, their altered expression can lead to related social phenotypes. This suggests that positive selection in this region of the genome contributed to the evolution of behavior during dog domestication by reducing fear and increasing tolerance to humans, thereby facilitating interspecies sociality [72].

At first glance there is a contradiction between the role of *Galnt17* gene in socialization in mouse and dogs. Mice with *Galnt17* KO show symptoms of ASD, including developmental delay, anxiety, etc. [65], while in dogs the TE-mediated downregulation of *GALNT17* expression is associated with increased sociability towards humans [17]. But it should be noted that in mice, researchers evaluated the socialization towards other animals of the same species, while in dogs the socialization towards humans was evaluated. Therefore, these two types of behaviors could differ significantly in their neurological and molecular basis, but this assumption requires further investigation. In addition, in mice it was gene KO while in dogs it was only reduced expression level. We cannot exclude that a complete loss of Galnt17 leads to a different behavioral phenotype as compared to reduced expression level. Also, it is possible that the role of Galnt17 in social behavior could be species-specific. Obviously additional research is required to fully clarify the contribution of Galnt17 to social behavior in dogs and other mammals. Generation of *Galnt17* gene KO animals of other species would help to clarify the role of Galnt17 in the regulation of social behavior in mammals. We propose that rat and rabbit models will be of particular interest since CRISPR/Cas9-mediated KO technology is well established in these species, these animals are small and easy to propagate, there are many established behavioral tests especially for rats and, finally, there is a possibility to compare the genetic variants in behavioral genes between domesticated and wild animals of these species.

## 4. Three-Dimensional Genome Alterations in the *WBSCR* and Social Behavior

Accumulating evidence suggests that alterations in 3D genome organization contribute to the development of human diseases [73]. TADs (0.1–1 Mb in size) and chromatin loops (0.05–0.5 Mb in size) are the basic units of the 3D genome architecture. They function at the level of genomic loci or individual genes by regulating contacts between genomic regions and their regulatory elements [73]. TAD boundaries harbor CTCF-binding sites and cohesin complexes, thereby insulating TADs [74]. The human genome contains both ultraconserved TADs that are found across multiple species- and human-specific TADs [74]. Importantly, TAD boundaries are enriched with retrotransposons that contribute to species-specific gene expression patterns. In addition, transposons as well as SNPs can disrupt 3D genome organization by deleting or inserting CTCF-binding sites, consequently altering TAD boundaries and chromatin looping [75].

Chromosomal aberrations (deletion, duplications, insertions) are the most frequent SVs that affect TAD and chromatin loop borders in the mammalian genome [73]. The predominant deletions/duplications in patients with WBS span approximately 1.5 Mb (Figure 1). Such large-scale alterations inevitably disrupt TAD boundaries and short-range chromatin interactions. Consequently, evaluating 3D genome organization within this locus in WBS and WBDS patients, compared to healthy individuals, is of paramount importance. Substantial evidence indicates that these extensive genome rearrangements perturb proper interactions between gene clusters and dysregulate the expression of neighboring genes, including *AUTS2* and *GALNT17*, which play the role in the regulation of behavioral phenotype.

The first evidence demonstrating the impact of large-scale chromosomal rearrangements in the *WBSCR* locus on activity of flanking genes were obtained by Merla et al. who showed significantly reduced expression levels of genes flanking the WBS deletion in skin fibroblasts and lymphoblastoid cells from WBS patients (e.g., *ASL*, *KCTD7* genes on centromeric side; *HIP1*, *POR* and *MDH2*—on telomeric side) [76]. Notably, some affected genes are located up to 6.5 Mb away from the deletion boundary (e.g., *KCTD7*, Figure 1). *AUTS2* expression was also reduced (though not statically significant), but *GALNT17* expression is not analyzed in this work [76]. Taken together these findings suggest that large deletion affects long-range cis-regulatory interaction in the *WBSCR*.

**Figure 2:**
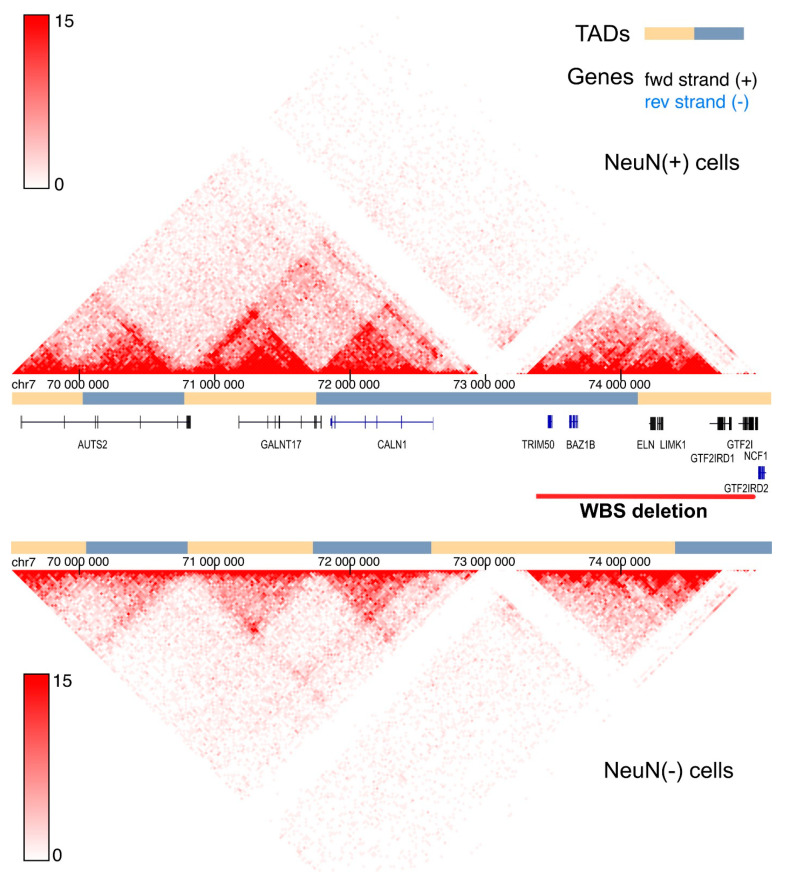
Schematic representation of the human Williams–Beuren Syndrome Critical Region (*WBSCR)* genomic locus integrated with the 3D Genome Browser [78] for Hi-C data exploration. The figure depicts the chromatin organization of the *WBSCR* genomic region in neuronal (NeuN(+)) and non-neuronal (NeuN(−)) cells. Hi-C data derived from cells of the left posterior superior temporal gyrus [79] are shown. The Hi-C interaction map displays a 6 Mb region on chromosome 7 (chr7: 69,500,000–75,500,000) at 25 kb resolution. Alternating yellow and blue bars demarcate predicted TADs.

Subsequent studies employing 3D genome analysis directly corroborated these initial observations. Using 4C-seq on lymphoblastoid cells from WBS patients versus healthy donors, interactions between the *WBSCR* locus and its flanking regions were dissected [77]. The interactions of *WBSCR* with genes flanking *WBSCR* on both sides were detected. Genes on the telomeric side (*HIP1*, *POR* and *MDH2*) primarily interacted with the region that included *ELN*, *LIMK1*, *EIF4H* and *CLIP2* genes. The closest gene at the telomeric side (*HIP1*) of the *WBSCR* was located 1 Mb away from the commonly deleted region (Figure 1). Genes from centromeric flank (*ASL*, *KCTD7* and *ZNF107*) which are located farther from the *WBSCR* as compared to *HIP1*, *POR* and *MDH2* showed weaker interaction with the *WBSCR* locus. In cells from WBS patients the interactions between *WBSCR* and other regions were diminished. Interestingly, *AUTS2* and *CALN1* were identified as interacting with both centromeric and telomeric genes [77]. *AUTS2* expression was significantly reduced in WBS cells as compared to healthy donor cells but *GALNT17* expression level was not assessed [77]. Importantly, *GALNT17* gene is located between *AUTS2* and *CALN1,* and these three genes span around 3 Mb in the human genome. Therefore, it is likely that these genes reside within distinct TADs (Figure 1 and Figure 2). Furthermore, transposon insertions in this region in dogs might affect TAD boundaries thus leading to perturbed expression of these genes, as exemplified by reduced *GALNT17* expression as a result of TE insertion [17].

To further support the importance of the 3D genome organization for proper expression of the genes in the *WBSCR*, Engmann et al. demonstrated a chromatin loop-mediated interaction between the *AUTS2* and *CALN1* genes [80]. Cocaine administration in rats disrupted CTCF binding and looping in this region thus inducing the higher expression of both genes [80].

Analysis of copy number variants (CNVs) in a large cohort of children with developmental delay identified highly recurrent deletions of human-specific TAD boundaries [74]. One of these boundaries is located within the *WBSCR* locus, 200 kb upstream *AUTS2* gene, between *GALNT17* and *AUTS2* [74]. CRISPR/Cas9-mediated deletion of this TAD boundary resulted in elevated *AUTS2* expression. Unfortunately, expression levels of *GALNT17* and genes typically deleted in WBS were not assessed in this study. It would be of great interest to determine if these genes are also affected by the deletion of human-specific TAD boundary in the *WBSCR* locus.

Relevant to this context, chromatin in neuronal cells forms more compact domains that constrain gene expression. Hi-C map of the *WBSCR* genomic region reveals stronger long-range interactions in neurons compared to other brain cells, with TADs also appearing more prominent (Figure 2). These interacting regions likely contain developmental transcription factors correlating with repressed status of many genes in neurons [79].

Taken together, recent findings utilizing 3D chromatin structure analysis provide crucial insights into the complex relationship between structural variants in the *WBSCR* locus and regulation of gene activity. This will have an impact on our understanding of the genetic basis of human neurobehavioral disorders. At the same time, a thorough and systematic analysis of the 3D genome regulation in the *WBSCR*—including the delineation of all TADs, loops and their boundaries across different cell lines, accompanied by the analysis of gene expression and histone modification—would be invaluable for understanding of many human pathologies, including ASD and drug addiction. Several symptoms of *AUTS2* syndrome and features of *Galnt17* KO mice are strikingly similar to those of WBS patients. These data unequivocally indicate that the phenotype of WBS patients, namely developmental delay, behavioral disturbances and ASD symptoms, at least in part are caused by dysregulated expression of *AUTS2* and *GALNT17*.

In addition to the effect on human neurobehavioral disorders recent work from vonHoldt’s lab for the first time showed that 3D genome changes are involved in the evolution of social behavior in dogs. This links animal domestication and human neurobehavioral disorders through the *WBSCR* genomic locus. As stated above, TEs are enriched at TAD boundaries [75], and there are several TE in the canine *WBSCR* locus that are associated with social behavior towards humans [68,69]. In particular these TEs were identified in the first intron of *GALNT17*, the fifth intron of *POM121* and the seventeenth intron of *GTF2I* [17]. The TE insertion in the intron of *GTF2I* represents an ancestral genotype of the gray wolf genome, and its presence is associated with diminished human-directed sociability [81].

Tandon et al. found that a SINE (Short-Interspersed Nucleotide Element) retrotransposon in intron 17 of canine *GTF2I* gene facilitates a chromatin loop formation, creating contact between introns 17 and 1 [81]. In the absence of the retrotransposon, the contact between the introns is not detected. At the molecular level the TE insertion does not affect the overall *GTF2I* transcript level but instead promotes the alternative splicing and increased usage of exon 18. This intronic interaction is likely mediated by the E2F1 transcription factor which binds to sequences within the TE in intron 17, facilitating contact with the first intron [81]. However, molecular details of this model require further experimental delineation. At the tissue level, transposon insertion in intron 17 induced differential expression of the extracellular matrix-related genes. The significance of these changes on social behavior remains unclear.

While the transposon insertion in *GTF2I* intron 17 is characteristic of the gray wolf genome, evolutionary selection in dogs appears to have favored absence of this TE. This provides the first evidence for chromatin structure influencing the evolution of social behavior in dogs [81]. As shown earlier by the same group, retrotransposons in the first intron of the *GALNT17* gene decrease its expression and increase sociability in dogs [17]. It will be interesting to find out how the presence of this retrotransposon in *GALNT17* gene affects the boundaries of TADs and loops and overall 3D chromatin structure.

Taken together, these results clearly indicated that the complex phenotype of neurodevelopmental disorders, such as WBS or WBDS, might arise not only from changes in copy number of gene but also from perturbed 3D genome organization in the locus. Therefore, investigating the relationship between structural variants of genes in the *WBSCR* locus and social behavior/domestication in mammalian species beyond dogs holds significant interest. We propose rats and rabbits as highly suitable model species for the evaluation of social behavior and generation of genome-edited animal models. These animal models will aim in the dissection of molecular basis of evolution of social behavior in animals and molecular mechanisms underlying human neurobehavioral disorders.

## 5. *GALNT17* (*WBSCR17*) Gene

Recent data from mouse models and analysis of genetic basis of domestication of dogs placed the *GALNT17* gene central to the regulation of social behavior in mammals. But our current knowledge on functions of Galnt17 is limited. In the following section we will summarize known cellular functions of Galnt17 as well as data from mouse models and genetic studies in humans that highlight putative functions of Galnt17 in different species.

### 5.1. Cellular Functions of Galnt17

*WBSCR17* gene was identified as one of the transcripts in the *WBSCR* locus (7q11.23) with high expression in brain and heart [82]. Later, on the basis of homology to human and rat *GALNT9* gene a novel gene named *GALNT17* was cloned and it was found identical to *WBSCR17* [83]. Detailed analysis of *GALNT17* transcription revealed its expression in cortex and cerebellum [83], thus indicating the role of Galnt17 protein in the development and functioning of the CNS. Human *GALNT17* gene is 580 kb long, contains 11 exons and encodes the single isoform of *N*-acetylgalactosaminyltransferase enzyme. Galnt family in human consists of 20 enzymes that catalyze transfer of *N*-acetylgalactosamine (GalNAc) from uridine-5′-diphospho-*N*-acetylgalactoseamine (UDP-GalNAc) to hydroxyl group of serine or threonine amino acid residues in proteins [84]. This type of post-translational modification is called *O*-glycosylation or mucin-type *O*-glycosylation since mucin family proteins contain multiple *O*-glycosylation sites and are the best studied *O*-glycosylated proteins. Galnt family enzymes play an important role in the regulation of multiple cellular processes in health and disease, including cancer [85]. Unlike many other Galnts, cellular functions and properties of Galnt17 are barely known.

**Figure 3:**
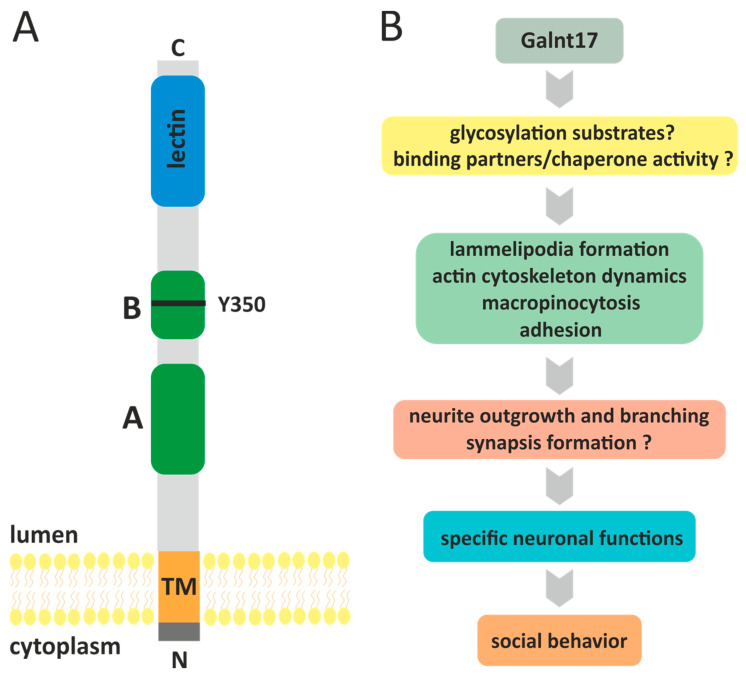
(**A**) Scheme of human Galnt17 protein. Galnt17 is a type II transmembrane protein that is anchored in the plasma membrane of Golgi apparatus by a 20 aa- long transmembrane domain (TM) with the C-terminal part of the protein facing the lumen and short *N*-terminal cytoplasmic tail (7 aa, dark gray). According to Uniprot (Q6IS24, Hs Galnt17), the catalytic domain is divided into subdomains A (151–262 aa) and B (319–381 aa), shown in green. GALNT motif (334–366 aa) is located within catalytic subdomain B. Ricin-like lectin domain (465–594 aa) is located at the C-terminus. Y350—a tyrosine residue within GALNT motif that is typical for Y-subfamily of GALNTs. (**B**) Diagram showing the putative role of Galnt17 in the regulation of social behavior in mammals. At the molecular level Galnt17 by glycosylating its substrates or binding to protein partners regulates cellular processes (e.g., actin cytoskeleton dynamics and adhesion) which are critical for proper neuron function in certain areas of brain thus specifically affecting social behavior of mammals.

Human Galnt17 is a type II transmembrane protein of 598 amino acids. Galnt17 is composed of a short *N*-terminal cytoplasmic tail of only 7 aa long, transmembrane domain of 20 aa and extracellular domain that includes GT1 motif, Galnt motif and lectin domain (Figure 3) [83,86]. Upon transient expression in HEK293 cells, Galnt17 is glycosylated and, like most of the Galnt family proteins, is localized to the Golgi complex [83]. This is in line with the notion that mucin-type *O*-glycosylation is induced in the Golgi complex and continues as glycosylated protein is translocated from cis- to trans-Golgi compartment [84]. Despite the high sequence similarity with Galnt family members, initially it was discovered that, unlike Galnt1, recombinant Galnt17 (w/o cytoplasmic and transmembrane domains) does not glycosylate peptide substrates derived from MUC1a, MUC5AC and MUC7 proteins [83].

In further studies, low activity of Galnt17 towards peptide substrates from MUC7, MUC5AC-1, MUC5AC-2 proteins, but not towards MUC1 or MUC5AC-3, was shown [86], indicating that Galnt17 is catalytically active. It is noteworthy that Galnt17, similarly to several the so-called Y-subfamily Galnts, contains amino acid substitution of the conserved tryptophane residue for tyrosine in the GALNT motif (W->Y) [86]. More specifically, prototypical Galnt1 enzyme contains two invariant tryptophane residues in the GALNT motif within catalytic domain (W316 and W328). Substitution of W328 with any amino acid renders the enzyme completely inactive, while substitution of W316 with aromatic amino acid, such as tyrosine, significantly, but not completely, reduced the catalytic activity of the enzyme [87]. Tryptophane residue in the W328 position of Galnt1 is invariant in all members of the Galnt family, while Galnt enzymes of the Y-subfamily (Galnt8, Galnt9, Galnt17, Galnt18) carry W->Y substitution in the position similar to W316 in Galnt1 [86,87]. Therefore, this substitution (Y350 in Galnt17) is likely to explain the low catalytic activity of Galnt17. Nevertheless, analysis of *O*-glycosylation profile in cells with Galnt17 knockdown revealed several differentially glycosylated proteins, thus indicating the existence of protein targets for Galnt17-mediated *O*-glycosylation [86]. Currently these putative Galnt17 targets are unknown as it is not clear whether the changes in the glycosylation profile are direct or indirect consequences of Galnt17 activity. It would be interesting to determine if the reverse substitution (Y350W) would increase the catalytic activity of Galnt17.

At the present time it is not clear what the mechanisms of Galnt17 functioning in cells are and, in particular, what is the significance of its low catalytic activity. Probably, Galnt17 performs certain tasks that are not related to its catalytic activity. For example, it was demonstrated that another Y-subfamily protein Galnt18 can act as a chaperone for other Galnts thus regulating the homeostasis of endoplasmic reticulum [88,89]. Thus, except for several in vitro-tested peptides, Galnt17 substrates as well as protein-binding partners are unknown.

Most data regarding cellular functions of Galnt17 were obtained by Nakayama et al. [86]. siRNA-mediated knockdown of *GALNT17* transcript in HEK293T cells cultured on fibronectin led to decrease in cell area, cell rounding, dissolution of lamellipodia, decrease in F-actin and paxillin (marker of focal contacts) staining [86]. These data allowed us to conclude that Galnt17 stimulates lamellipodia formation and promotes adhesive phenotype. Lamellipodia plays an important role in the regulation of cellular morphology, adhesion and motility [90]. Formation of lamellipodia and filopodia lies at the basis of neurite outgrowth, axon guidance and branching thus allowing for generation of novel synaptic contacts [91,92]. It is interesting to note that, similarly to Galnt17, Auts2 protein regulates formation of lamellipodia and filopodia, cell motility and neurite outgrowth [93]. Cellular functions of these two proteins seem to be tightly connected to the development of contact between neurons.

Accordingly, it might be speculated that cognitive and behavioral disturbances in model animals with *Galnt17* KO and in patients with WBS are the consequences of impaired formation of neuronal contacts due to Galnt17 dysfunction. However, the exact role of Galnt17 in the regulation of actin cytoskeleton dynamics in neurons and the contribution of *Galnt17* KO/knockdown to the development of the behavioral phenotypes are not known. It should be noted that so far, the effects of Galnt17 knockdown have not been evaluated in neuronal cell lines. It would be interesting to find out how Galnt17 knockdown affects actin cytoskeleton and neurite outgrowth in neuronal cell lines or in primary neurons. In support of the Galnt17 role in the regulation of neurite formation, Nakayama et al. have shown that Galnt17 might be involved in axonal outgrowth in zebrafish brain [94] and, in *Galnt17* KO mice, abnormal development of axons and dendrites in Purkinje cells in cerebellum is observed [65].

Besides lamellipodia formation, Galnt17 is involved in the regulation of endocytosis. In one of the pioneer works, *GALNT17* gene was identified as a candidate gene that regulates endocytosis, cell proliferation and *N*-glycan branching [95]. Indeed, it was later shown that Galnt17 negatively regulates micropinocytosis and upon Galnt17 knockdown cells accumulated enlarged macropinocytic vesicles, although Galnt17 is not likely to affect clathrin/caveolin-mediated endocytosis [86]. High concentration of *N*-acetylglucoseamine (GlcNAc) increases *GALNT17* gene expression [86,95], that in turn can suppress micropinocytosis via Galnt17-mediated *O*-glycosylation of unknown substrates. The relationship between GlcNAc-induced *GALNT17* expression and regulation of micropinocytosis warrants further studies.

### 5.2. Galnt17 Knockout Mouse

Most of the data on functions of Galnt17 at the level of organism were derived from the analysis of *Galnt17* KO mice [65] that were generated by inserting the selective expression cassette into the first exon of *Galnt17* gene thus disrupting its expression. Transcript-level analysis in the hippocampus and cerebellum of homozygous *Galnt17* KO animals revealed a near-complete absence of the transcript. These data confirm the presence of only one transcript variant of this gene. Authors observed lethality of the homozygous KO mice at the C57BL/6J genetic background, but not at the hybrid C57BL/6J X C3H/He background. Reason for the observed lethality requires further investigation.

It turned out that many of the phenotype features in mice with reciprocal translocation with breakage site between *Auts2* and *Galnt17* genes [62] are recapitulated in *Galnt17* KO mice [65]. Developmental delay, particularly in the early neonatal period (lower body weight, delayed eye opening) was one of the prominent phenotypic features of the homozygous *Galnt17* knockout mice. Heterozygous knockout animals also exhibit developmental delay in the early neonatal period, which is compensated for with age. Homozygous knockout animals exhibit motor coordination problems, decreased social and exploratory activity while maintaining normal memory and learning ability, and abnormalities in the structure of the cerebellar vermis.

At the molecular level, RNA-seq identified many differentially expressed genes in the cerebellum of knockout animals; in particular, the largest number of downregulated genes belonged to the functional categories/terms that included neuronal differentiation, nervous system development, axon guidance, synaptic organization, cholinergic signaling and heparan sulfate synthesis that is required for axon guidance [65]. Downregulation of *Mid1* and *Folr1* gene might explain vermis development anomalies in the KO animals. Upregulated pathways included apoptotic signaling and integrin biosynthesis. Accordingly, TUNEL assay revealed increased apoptotic granular cells in vermis [65].

Reduced intensity of mucin-type *O*-glycosylated protein bands on lectin-probed blots of cerebellar protein extracts from *Galnt17^−/−^* animals clearly indicate that there are direct or indirect Galnt17 glycosylation targets in cells. Despite some efforts these proteins have not been identified so far.

Summarizing, *GALNT17* gene is not deleted in WBS patients and its role in the development of WBS is not clear. On the other hand, observed behavioral and social phenotypes of the *Galnt17* KO mice at least in part are similar to those seen in WBS and AUTS2 syndrome patients. These data imply that dysregulation of *GALNT17* gene expression might contribute to the development of symptoms of these disorders. In support of this idea, it was shown that mouse translocation that affect *Auts2* and *Galnt17* genes leads to the significant downregulation of their transcripts. Therefore, one might propose that in WBS or WBDS patients, as a result of large chromosomal reengagements, the boundaries of chromatin loops and/or TADs are dramatically changed thus leading to abnormal downregulation of *GALNT17/AUTS2* transcriptional activity. This hypothesis is supported by the evidence of *AUTS2* downregulation in WBS patients [76], but unfortunately none of the previous studies have addressed the expression level of *GALNT17* mRNA [74,76,77,80].

### 5.3. Galnt17, Human Diseases and Phenotypic Traits in Animals

Genetic studies allowed us to identify several SNPs that link *GALNT17* with human diseases or phenotypic traits in animals. Large-scale GWAS identified novel SNP rs9638616:T in intron of *GALNT17* gene in Asian populations that was associated with development of PD [64,96]. This SNP is associated with white matter tract and functional connectivity dysfunction in the supplementary motor area in cortex [97] and is located near miRNA genes in *GALNT17* gene intron. Therefore, it is not clear whether the increased risk of PD development is associated with dysregulation of *GALNT17* gene or miRNAs. Another intronic SNP rs17058752 in *GALNT17* gene is associated with the development of age-related cataract in the Korean population [98]. Finally, in human, *GALNT17* gene is differentially methylated in breast cancer and is included in seven gene signatures that allow us to distinguish between low- and high-risk breast cancers [99]. Significance of this finding is not clear since normally *GALNT17* is not expressed in the mammary gland. Further studies are required to elucidate the role of *GALNT17* methylation/expression in breast cancer.

In cows, SNP in *GALNT17* gene was identified as associated with milk oligosaccharides synthesis [100] which has a clear connection to enzymatic function of Galnt17. Expression of *GALNT17* gene is downregulated in the endometrium of pregnant cows, but significance of this observation is unknown [101]. In addition, Galnt17 protein level is increased in serum of rats 2 weeks after traumatic brain injury [102]. These data suggest that Galnt17 might have protective/regenerative role in the CNS. Based on the previous finding it is tempting to speculate that Galnt17 is increased in order to promote axon outgrowth, guidance and formation of synaptic contacts to follow the injury, but on the other hand the increase in Galnt17 might reflect the release of the protein from injured brain cells. Both hypotheses require further experimental confirmation.

Finally, circular RNA circ_*WBSCR*17 (circ_0080425, located in the fifth exon of *GALNT17*) is significantly upregulated in a mouse model of a diabetic nephropathy and functions as a pathogenic circular RNA acting as a molecular sponge for miR-185-5p, preventing the microRNA from binding to its target *SOX6*. As a result, *SOX6* is overexpressed to promote inflammatory responses and fibrosis in kidney cells by inhibiting cell proliferation and promoting apoptosis [103]. Molecular mechanism of circ_*WBSCR*17 should be clarified in further studies.

To briefly summarize, Gant17 is an emerging player in the field of social behavior in animals and neurodevelopmental disorders in humans. Preliminary observations from GWAS and other genetic studies indicate that *GALNT17* gene could be associated with CNS diseases in humans and with phenotypic traits in animals. These data further emphasize the importance of dissecting molecular mechanisms of both *GALNT17* gene regulation and Galnt17 protein activity in cells. In mouse *Galnt17* knockout leads to behavioral abnormalities and symptoms of autism spectrum. At the molecular level functions of Galnt17 are far from being understood and the relationship between ASD phenotype and the loss of Galnt17 are not entirely clear. Galnt17 regulates lamellipodia formation, cell adhesion and micropinocytosis in HEK293T cells but its glycosylation substrates and/or protein binding partners are not known. A plethora of genes are differentially expressed in cerebellum of *Galnt17* KO mice and several proteins are differentially glycosylated but exact functions of Galnt17 in neuronal cells have not been investigated in detail so far. Recent advances in the dissection of genetic basis of dog’s domestication place Galnt17 central to social behaviors in dogs. Taken together the recent data suggest that *GALNT17* is an attractive target for genome editing for the purpose of changing social behavior in animals. Besides that, it is of a great interest to dissect the molecular basis of Galnt17 functions in cells, and, in particular, in CNS.

## 6. Conclusions

**Figure 4:**
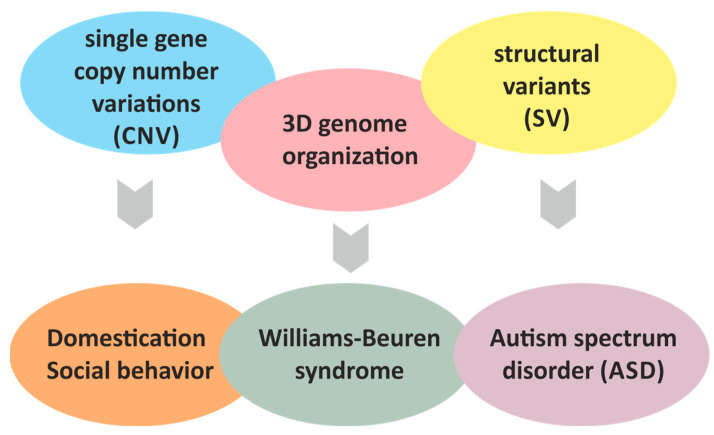
Scheme of the putative relationships between changes in copy number variants (CNVs), structural variants (SVs) and 3D genome organization in the Williams–Beuren Syndrome Critical Region (*WBSCR*) locus and domestication and neurobehavioral disorders.

Domestication is a multifaceted process, with its genetic foundations often being polygenic and not entirely clear. The primary indicator of successful domestication is the animal’s friendliness towards humans. The quest to identify the genetic basis of this behavioral trait in animals led to the discovery of the *WBSCR* locus, a deletion of which in humans causes WBS. However, deleting an entire locus in humans not only alters the behavioral phenotype but also causes a range of severe physical health issues. Therefore, finding individual genes that are crucial for domestication without affecting physical health is a highly promising and urgent task. One such candidate gene is the *GALNT17*. In particular, it has been found that certain polymorphisms in this gene differ between domestic dogs and wolves. The molecular pathways in which the *GALNT17* gene product participates are not well-known, but this gene may be considered as a potential target for genome editing in domesticated animals to achieve the desired phenotype. A body of experimental data suggest that copy number changes and structural variants in the *WBSCR* affect the gene regulations directly as well as through alteration of 3D genome organization thus affecting both domestication of animals and development of neurobehavioral disorders in human (Figure 4). We emphasize the importance of further research aimed at unraveling the complexity of the relationships between genetic variants in the *WBSCR* and phenotypic traits in different animal species and in human. Advanced 3D genome analysis techniques and genome editing technologies are the methods of choice for solving these tasks.

## Data Availability

Data are contained within the article.

## Abbreviations

The following abbreviations are used in this manuscript:

ASD	Autism Spectrum Disorder
CNS	Central nervous system
CNV	Copy Number Variant
GalNAc	*N*-acetylgalactosamine
GlcNAc	*N*-acetylglucoseamine
GWAS	Genome-wide association studies
iPSC	induced pluripotent stem cell
KO	knockout
LCR	low-copy repeat elements
NCC	neural crest cell
PD	Parkinson disease
SINE	Short-Interspersed Nucleotide Element
SNP	single-nucleotide polymorphism
SV	structural variant
TAD	topology associated domain
TE	transposon element
UDP-GalNAc	uridine-5′-diphospho-*N*-acetylgalactoseamine
WBDS	Williams–Beuren region duplication syndrome
WBS	Williams–Beuren syndrome
*WBSCR*	Williams–Beuren syndrome control region
WSTF	Williams syndrome transcription factor
